# Receipt for a Deep Black Ink

**Published:** 1864-01

**Authors:** 


*Receipt for a Deep Black Ink.*—The *Chemical News* gives the following recipe, which may be useful to our country readers: Take 14 ounces of powdered galls, 5 ounces of powdered gum Senegal, 6 quarts of distilled or rain water, 6 ounces of liquor of green vitriol free from copper, 1 drachm of liquor of ammonia and 8 ounces of spirit of wine; mix these in an open vessel, and allow them to stand, stirring frequently, until the ink attains the desired blackness. This ink will not corrode steel pens.

